# Word Diffusion and Climate Science

**DOI:** 10.1371/journal.pone.0047966

**Published:** 2012-11-07

**Authors:** R. Alexander Bentley, Philip Garnett, Michael J. O'Brien, William A. Brock

**Affiliations:** 1 Department of Archaeology and Anthropology, University of Bristol, Bristol, United Kingdom; 2 Department of Anthropology, Durham University, Durham, United Kingdom; 3 Department of Anthropology, University of Missouri, Columbia, Missouri, United States of America; 4 University of Missouri, Columbia, Missouri, United States of America; 5 Department of of Economics, University of Wisconsin, Madison, Wisconsin, United States of America; Technical University of Denmark, Denmark

## Abstract

As public and political debates often demonstrate, a substantial disjoint can exist between the findings of science and the impact it has on the public. Using climate-change science as a case example, we reconsider the role of scientists in the information-dissemination process, our hypothesis being that important keywords used in climate science follow “boom and bust” fashion cycles in public usage. Representing this public usage through extraordinary new data on word frequencies in books published up to the year 2008, we show that a classic two-parameter social-diffusion model closely fits the comings and goings of many keywords over generational or longer time scales. We suggest that the fashions of word usage contributes an empirical, possibly regular, correlate to the impact of climate science on society.

## Introduction

For over a decade, leading scientific organizations such as the American Association for the Advancement of Science (AAAS), the Intergovernmental Panel on Climate Change, the American Geophysical Union, the National Academy of Sciences (NAS), and the American Meteorological Society have sent clear signals that Earth's climate is warming and that the changes are in large part the result of anthropic activities. Despite debate over precise mechanisms and the amount of warming brought on by various processes [1], scientific reports collectively demonstrate that “most of the observed warming of the last 50 years is likely to have been due to the increase in greenhouse gas concentrations” [2].

Despite the play these findings receive in the media and in venues organized by scientific bodies such as the AAAS, the response in terms of public opinion and behavior has been slow. Although there are substantial issues concerning the public trust in science [3], [4], as well as a widely held perception that climate change is only a distant threat [5], probably the underlying reason has to do with poor communication [6], [7] and “the role of language (metaphors, words, strategies, frames and narratives) in conveying climate change issues to stakeholders” [8]. Some of this concern focuses on journalists, whose regular use of terms such as “global warming” might be perceived as biased, whereas another concern focuses on climate scientists and specialized jargon that fails to convey key concepts [9].

Even the most well-intentioned communication approaches typically assume that the public consists of empty vessels “waiting to be filled with useful information upon which they will then rationally act” [8]. The shortcoming of this “information deficit model,” whereby ordinary people are simply supplied with expert information, is in neglecting social learning. People clearly share with each other their impressions of climate change and policy [10]. As they recognize this, policymakers are shifting from traditional information campaigns toward a more flexible ability to respond to these movements or at least trying to “nudge” them in certain directions [11].

As George Orwell famously reasoned [12], the stylistic use of language is central to political discourse. For just one documented example, opponents of the estate tax help influence attitudes in their favor by calling it a “death tax,” which magnifies the prospect of upward mobility [13]. Since climate science too is political, these dynamics matter, as certain trends of language use could lock the public into specific ways of defining, thinking, or interpreting climate change [8].

In our study below, we present a starting point for an empirical study of scientific “impact” as reflected by wider discourse. Our hypothesis is that certain keywords used in climate science will follow a distinct “boom and bust” fashion wave in general usage (distinct from the more specific usage in science), which can be modeled with a simple two-parameter logistic growth model. We fit the model to the word-frequency data using a simple statistical testing procedure [14] that minimizes the least-squared regression between the model and data over the space of the three input parameters. We then discuss how the fitting of this classic two-parameter social-diffusion model to the word data could contribute an empirical correlate to the impact of climate science on the public.

### Modeling language fashions in climate science

We aim to investigate general usage of climate-science vocabulary through the new “Ngram” database [15], which at present scans through over five million books published in seven languages since the 1500s (about 4% of all books), although Google recommends using data after 1800 for quantitative analysis (the sample before 1800 being very rare books). Using these remarkable new data, we can evaluate the evolutionary history of word frequencies to characterize the effective degree of fashion versus independent decisions to use a particular word or phrase [16]–[23].

For our case study focused on keywords used in climate science, we benefit from the study of Li *et* *al*. [24], who have already listed the top keywords for the period 2004–2009, the 1-grams among which include: *adaptation, biodiversity, climate, diatoms, drought, global, Holocene, isotopes, paleoclimate, phenology, photosynthesis, pollen, precipitation*, and *temperature*. As these represent important keywords in the narrow sphere of academic climate science, our aim is to investigate possible social-diffusion trends in more general usage of these words, via the much larger Ngram database.

We approach this with a simple diffusion model that would characterize word-frequency evolution along a continuum governed by two parameters, often interpreted to represent individual decision versus social fashion [20], [25]–[29]. The classic formulation of Bass [30] expressed at time 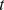 is
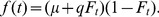
(1)


The first half of 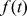 in equation (1) models the probability a word is used at time 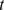 as proportional to its cumulative fraction, 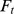, of all the times the word will eventually be used, as governed by the constant 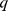. The second constant, 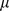, governs the relative rate of independent discovery (more detail in Methods).

In order to estimate the parameters of equation (1) to fit a data series, a useful formulation [31] would represent the cumulative number of times a word 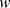 is used, 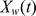 by
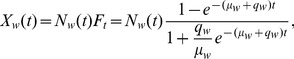
(2)where integer 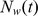 is the maximum number of times the word could have possibly appeared by time 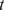.

Our aim is to fit the popularity of each word over time to the process described in equation (2). As the number of books grows with time, we need a dynamic 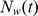 in equation (2) that allows the total potential number of times, 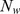, that the word could be used to increase with time accordingly. One approach is to allow 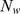 to grow in some predictable fashion over time, perhaps exponential growth,
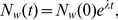
(3)where 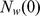 is a constant specific to word 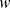 and 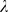 is a universal constant derived from the entire Ngram dataset. This approach, which we will call Model 1, substitutes 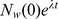 into equation (2) for the amplitude 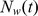:



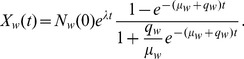
(4)To represent the number of word usages per year, rather than cumulative usage, we apply Model 1 as a difference equation, 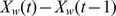, yielding.
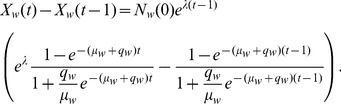
(5)


If the approximation of (3) for the total number of words is too crude, then a more data-driven approach we can explore, which we will call Model 2, is to assume that 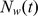 is some fixed fraction, 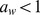, of the use of the word *the*:
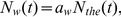
(6)where 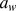 is a parameter specific to word 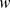. We then substitute 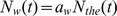 into equation (2), such that the difference equation, 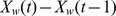, for Model 2 is



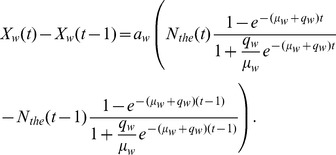
(7)For this alternative approach to the amplitude, the cumulative word counts of the word *the* (since 1800) produce the time series for 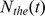. We propose that it is better to normalize to *the*, the most common word in English, than to use the gross total of Ngrams per year, because the full, unfiltered Google record includes growing numbers of characters, data, and other non-English “noise” over the past centuries.

In comparing the Bass diffusion model to the word data, we acknowledge that the parameter 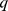 does not necessarily have to be “social,” as S-curves of adoption can be generated through individual learning in successive stages [29], and we show a simple “nonsocial” version of the model in our Methods. Because we are dealing with language, however, we maintain that the usefulness of a word depends intrinsically on how other people have used it. We therefore feel comfortable referring to the parameter 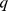 as the social parameter.

In any case, setting aside the epistemology of the meaning of 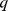, our aims are practical. To determine the amplitude term for Model 1, we start by finding a universal exponent 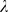 for the general growth equation (3) to fit the overall Ngram database. For each word 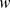 in our case study, we then seek the best values of 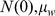, and 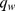 that lead Model 1 to fit its Ngram count through time. Alternatively, for Model 2, we seek the best values of 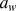, 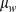, and 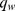 to fit the Ngram count for the word through time, where the amplitude is governed by a fraction, 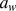, of cumulative usage of *the* through time.

The modified Bass model from equation (2), applied as a difference equation via equation (5) for Model 1 or equation (7) for Model 2, can be fitted to to the yearly usage counts for each of the individual words. To fit the model to the data for each word, we optimize the word-specific values of 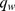 and 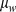, plus either 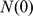 for Model 1 or 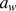 for Model 2. For this study, we eyeball the start date of the diffusion curves, which is actually very effective (we discuss below how this might be systematized).

## Results

**Figure 1:**
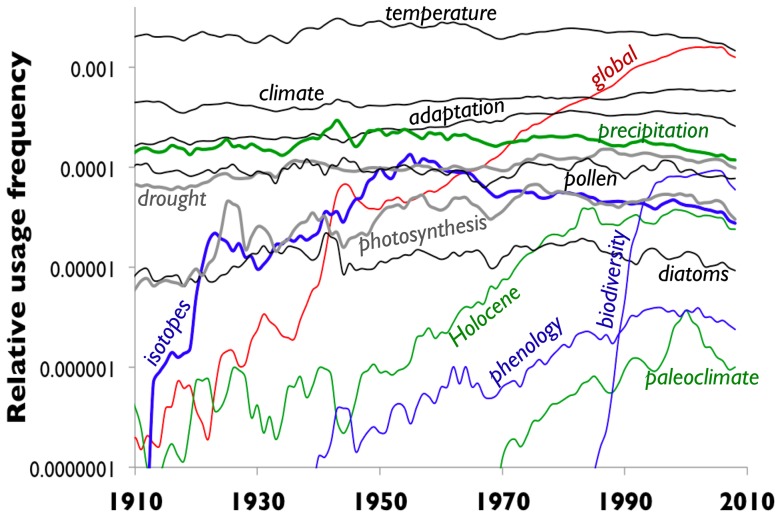
The popularities of the top climate change 1-grams in the Google Ngrams database, normalized to the word *the* and using a logarithmic scale. Shown here is the last century of public usage of a set of the top climate-change keywords in recent scientific publications [24], which include: *adaptation, biodiversity, climate, diatoms, drought, global, Holocene, isotopes, paleoclimate, phenology, photosynthesis, pollen, precipitation*, and *temperature*.

We extracted the use statistics from the Google database for the 1-grams among the top keywords used in climate science (but not the 2-grams, such as *climate science*). Figure 1 shows the popularities (logarithmic scale) of these climate-science words since 1900. Among the sample, the words that show relatively steady rate of use include *climate*, *diatoms* and *pollen* (Figure 1). These words can be predicted by Model 1 or Model 2, but in the trivial sense that the social parameter 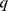 is very small or zero (Table 1).

**Table 1:** Best-fit values to the yearly word frequencies from the Google 1-gram database.

Fit by Model 1	Start year	*N*(0)	σ(*N*)	*μ*	σ(*μ*)	*q*	σ(*q*)
paleoclimate	1969	3,752	3,226	0.00000016	n.d.	0.48	0.39
biodiversity	1984	319,760	283,310	0.002	0.00070	0.27	0.22
global	1943	2,470,300	2,248,900	0.0000067	0.00000035	0.17	0.15
phenology	1973	13,679	10,954	0.0046	0.0018	0.12	0.08
Holocene	1945	82,010	56,102	0.0011	0.00013	0.08	0.04
isotopes	1931	77,827	74,464	0.00094	0.00021	0.21	0.18
photosynthesis	1900	64,075,000	n.d.	0.0000052	n.d.	0.00079	n.d
adaptation	1800	1,668,000	n.d.	0.000016	n.d.	0.0078	n.d.
precipitation	1890	17,425,000	n.d.	0.00037	n.d.	n.d.	n.d.
temperature	1860	31,800,000	n.d.	0.00084	n.d.	n.d.	n.d.
drought	1950	1,870,500	n.d.	0.0032	n.d.	n.d.	n.d.
diatoms	1870	2,945,900	n.d.	0.000029	n.d.	n.d.	n.d.
climate	1943	97,375,000	n.d.	0.00010	n.d.	0.015	n.d.
pollen	1860	2,451,900	n.d.	0.00056	n.d.	n.d.	n.d.

Fit by Model 2	Start year	*a*	σ(*a*)	*μ*	σ(*μ*)	*q*	σ(*q*)
paleoclimate	1969	0.0000006	0.0000005	0.00000005	n.d.	0.53	0.43
biodiversity	1984	0.000033	0.000029	0.0015	0.0004	0.277	0.225
global	1943	0.00077	0.00072	0.00000078	n.d.	0.18	0.16
phenology	1973	0.0000018	0.0000015	0.0037	0.0012	0.14	0.10
Holocene	1945	0.000021	0.000018	0.00063	0.00029	0.10	0.07
isotopes	1931	0.000036	0.000035	0.0026	0.0012	0.15	0.13
photosynthesis	1900	0.000039	0.000036	0.0011	0.0002	0.054	0.040
adaptation	1800	0.0465	n.d.9	0.000018	n.d.	0.037	n.d.
precipitation	1890	0.00014	0.00014	0.0042	0.0022	0.042	0.031
temperature	1860	0.0019	0.0018	0.0041	0.0018	0.032	0.021
drought	1950	0.00011	0.000098	0.011	0.009	0.029	0.016
diatoms	1870	0.000011	0.000010	0.00096	0.00005	0.045	0.032
climate	1943	0.015	n.d.	0.00034	n.d.	0.0052	n.d.
pollen	1860	0.000088	0.000080	0.017	0.0052	0.0037	n.d.

The listed 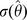 values yield the 95% confidence interval, i.e., from 
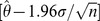
 to 
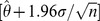
 for the nonlinear least-squares estimates of each parameter 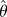 in the previous column (assuming normally distributed errors).

The start date indicates the first year of the time series, which was estimated to be the start of the Bass curve. Errors on the parameters were calculated except where “n.d.” indicates that, in the fitting process, the model is insensitive to this parameter.

Eight of the words, in contrast, demonstrate a Bass-like wave — *biodiversity, global, Holocene, isotopes, phenology*, and *paleoclimate* on a time scale of decades and *precipitation, photosynthesis*, and *adaptation* at a century time scale. These waves begin at different times, from the late 19th century to the late 20th century, but occur on a range of different timescales (Figure 1).

**Figure 2:**
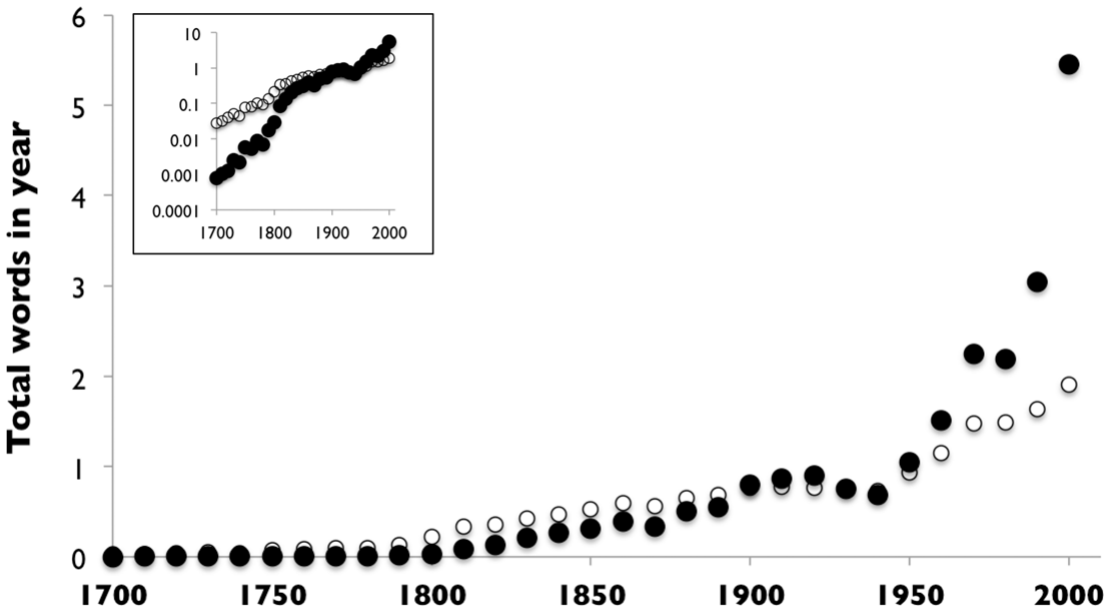
Total number of word usages per year recorded by the Google database, in billions. Inset shows the same data with logarithmic y-axis.

Using equation (3) for the amplitude term for Model 1, we see from the entire Google 1-gram database that the number of words published, 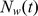, grew fairly smoothly for three centuries, by about 3% per year (Figure 2). There were 793,000 words for the year 1700, which grew to 5.46 trillion words for the books of 2000. The number of words in each year of the record fits an exponential growth function proportional to 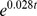.

**Figure 3:**
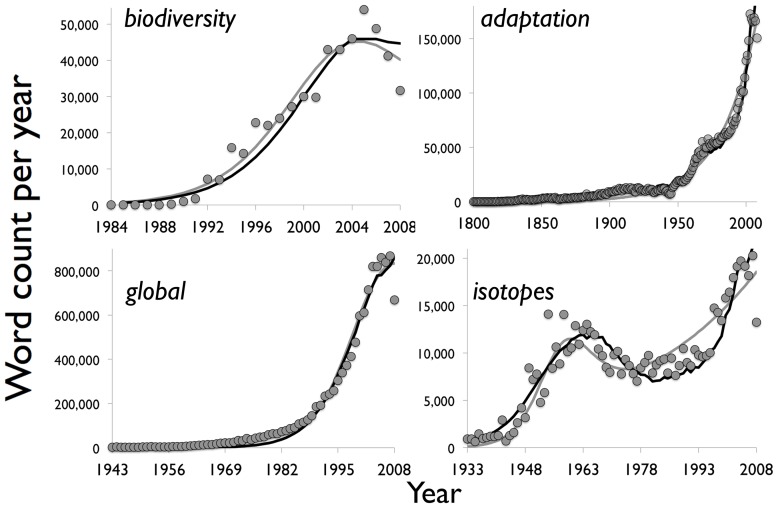
Word counts per year versus Model 1 and Model 2, for selected words as examples. Gray circles show the word data, the gray curve shows Model 1, and the black curve shows Model 2 (occasionally the black curve obscures the gray curve). Plugging in the best-fit values of 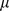, 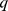, and 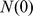 from Table 1 (top half) for each word, Model 1 uses equation (5) to represent the word-usage rate. For Model 2, we plug the word-specific values of 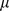, 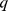, and 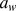 from Table 1 (bottom half) into equation (7).



Applying this to equation (5), we let 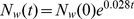. Using this expression for exponential growth in amplitude in Model 1, the gray curves in Figure 3 show the best fit of equation (5) to the yearly word count of four words from the list: *biodiversity, global, isotopes*, and *adaptation*. Table 1 lists the best-fit parameters 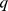, 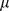, and 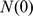 under Model 1 for the full list of words. For example, plugging in the specific values of 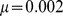, 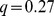, and 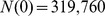 from Table 1 for *biodiversity*, and with 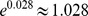, the Model 1 difference equation (5) is

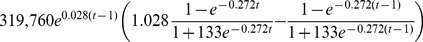
(8)usages of *biodiversity* per year 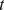 (Figure 3). The three other Model 1 curves in Figure 3 are similarly produced by plugging the corresponding parameter values for the word (top half of Table 1) into equation (5).

We then explore the alternative approach of Model 2, which uses the actual yearly counts of the word *the* for the amplitude term of equation (7). The Model 2 results fit the individual words better than Model 1 (Figure 3, black curves), yielding better estimates of confidence intervals around the parameters **in** Table 1). Each Model 2 curve in Figure 3 is produced by plugging the specific parameter values 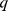, 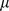, and 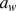 for the word (bottom half of Table 1) into equation (7). Taking *biodiversity* again as an example, we plug in its specific values of 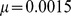, 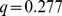, and 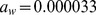 from Table 1, so that the Model 2 difference equation (7) is
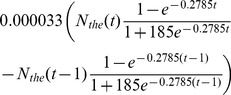
(9)usages of *biodiversity* per year 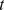.

As we see in Figure 3, the raw word count of each word is underlain by the exponential growth in published English over the years. The raw yearly counts for a word rarely return to zero, because the exponential growth in amplitude dominates as 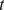 increases. Among our examples in Figure 3, this can be seen particularly well for the word *isotopes*, where the ‘Bass’ part of Model 1 yields the first peak by midcentury, but then the exponential growth in amplitude dominates by later in the century.

**Figure 4:**
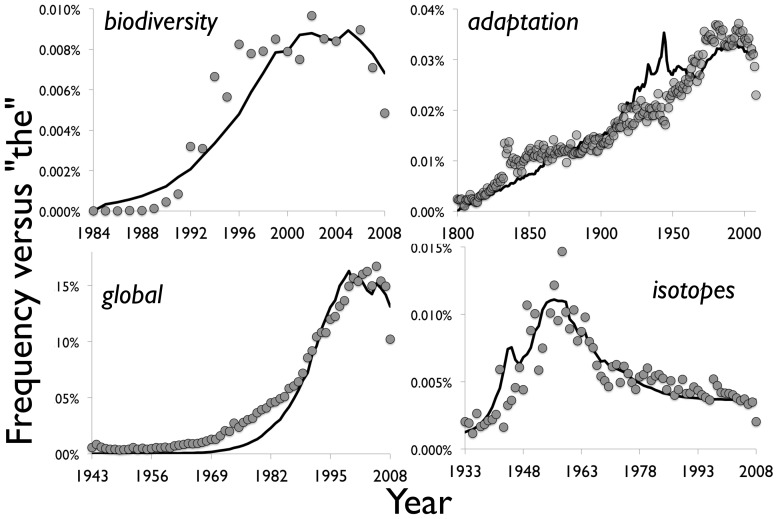
Normalized word counts per year versus normalized Model 2. Shown are the word data from Figure 3 fitted by Model 2, each normalized by the yearly count of the word *the* in the Google database.

Hence the raw count does not convey very well how most of these words ultimately decline in their relative frequency among all words. Rather than try to second-guess when this exponential growth in total word count will level off (which is even more ambiguous now with digital publishing), we simply present the same results normalized by the counts of *the* in Figure 4. The normalized plots in Figure 4 show the decline in relative frequency after the peak, as well as subtler changes. When we normalize *isotopes*, for example, the curve has just the one major peak in midcentury (Figure 4). The other Model 2 curves in Figure 3 are shown in black, plugging the corresponding parameters from the bottom half of Table 1 into equation (7).

Looking in more detail at these fits, we recognize that the probabilities 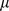 and 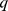 cannot be expected to be uniform over time and different communities. If we assume that their mean values remain the same over time, we can introduce “noise” in both 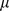 and 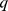 during these modeled dynamics (detailed in Methods). Using maximum likelihood to find the parameters of best fit to each word diffusion, we can then measure the errors (residuals) as a function of time to evaluate the predictions of the noisy Bass model.

**Figure 5:**
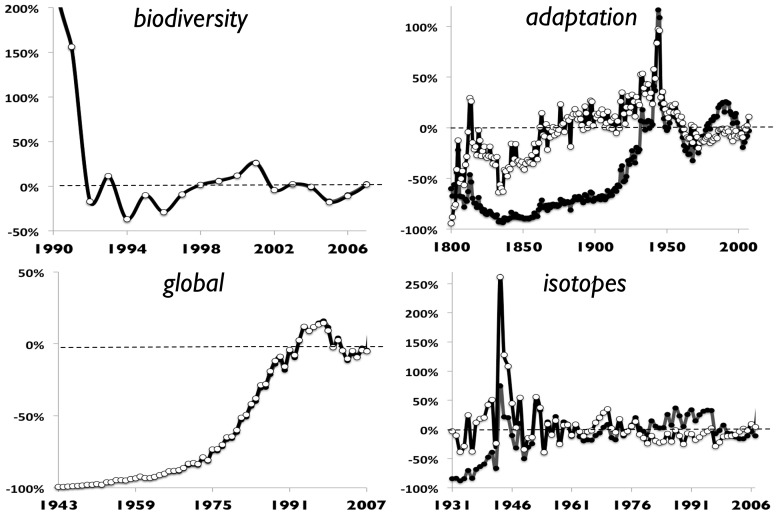
Residuals from the best-fit Model 1 and Model 2, expressed as percentages of the actual frequency of each word through time. Examples shown are *biodiversity, global, adaptation*, and *isotopes*. Filled circles for Model 1, and white circles for Model 2 (results overlap substantially for *biodiversity* and *global*).

To evaluate the noise predictions, we consider how the actual word frequency departs from the model over time for each word in our example set. It is instructive, therefore, to treat the fitted diffusion model as the null model and then plot the departures from this null over time. We measure these departures simply by taking the difference between the prediction of the model and the actual word count for each year, and then express this as a fraction of the actual word count. Figure 5 illustrates departures for several examples; note that the magnitude of the residuals decreases over the long term for *biodiversity*, *adaptation*, *global* and *isotopes*. This suggests the noise is more in 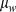 than in 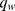. Indeed, we generally found the fitting of 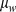, which varies by orders of magnitude among our examples, more difficult than fitting 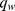, which is more consistent (Table 1).

Interestingly, the residuals for *global* and *isotopes* increase at the very end of the time series (just year 2008), due to a faster drop in real frequency compared to the model prediction. We do not show the 2008 residuals in Figure 5, however, because we suspect this may be an ‘edge effect’ in the datasets at year 2008, when the Google Ngram count is truncated, but perhaps they suggest some learning bias against these two words by 2008. Only more data in the future can answer this question.

## Discussion

We have found that the same classic two-parameter Bass model closely fits the usage of certain scientific keywords in the more general, public sphere of all published books. Among the two approaches to the amplitude portion of the model, the more accurate is to use the actual observed number of uses of the word *the* per year as an input parameter, compared to the coarser estimate of a purely exponential growth in the number of words through time.

Because the scale of these keyword trends varies from centuries to years, we posit that the explanation is not a normal distribution of independent response times but rather the diffusion of these words through social learning. Several of the words conform to the suggestion that there is a typical diffusion time of about 30–50 years, or a timescale “roughly equal to the characteristic human generational time scale” [32]. A few words however, such as *adaptation, precipitation, photosynthesis*, and possibly *temperature*, appear to be diffusing on a scale of multiple generations. One difference, which may be important, is that we studied selected popular words that diffused en route to becoming popular, whereas Petersen et al. [32] looked at all words above a certain minimal threshold of usage, the majority of which may never have become popular. Future studies might explore whether there is a certain threshold of popularity where these lifespan dynamics change [16], [33].

These diffusions are visible in *general* usage, and so we are not suggesting that climate science itself is a fashion. We suggest that some of the core vocabulary of climate science becomes passé in public usage, even as the scientific activity may remain steady. A new keyword database of scientific discourse (arxiv.culturomics.org) shows the usage of these climate-science keywords in science does not show the same marked social-diffusion curves that we find in public/general usage represented by the Google Ngram database. This bears consideration as a factor (among the clear economic and other barriers) for why the social and political impact of the convincing climate evidence has been disappointing.

The model is widely applicable. In fact, our original motivation for this case study was in observing that the simple model of equation (2) fits the coming and going of many of the fashionable words that Michel and colleagues [15] used as examples. There clearly appear to be words with high 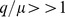, which rise and fall as symmetric waves, such as *feminism* or *global*. Also, there are words with low 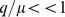, which rise very quickly after an event and then decline exponentially. The best examples of this are the names of a calendar year (“1883,” “1910,” “1950”), which follow the low 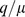 pattern, starting just after the named calendar year [15]. Some words rise with good fit to the social-diffusion pattern but then persist without declining, presumably because they acquire a basic function in the language. These include useful technologies or scientific discoveries, such as *DNA*, *telephone*, and *radio* [15]. The word *radio*, for example, shows a fashionable rise during the initial stage but then settles into the more stable, functional stage.

The Bass model we adapted in this study has been used effectively for decades in marketing and other applications to capture social versus independent spread of purchases of consumer goods, adoption of technologies, and more recently in online media [34]. As has been suggested for other public-communication concerns, such as recent flu scares [18], we suggest that the three-parameter social-diffusion model can be a highly useful tool for getting a quick, rough assessment of how words are chosen and shared within discourse, whether published in academic journals, reported by the media, or found during online searches or on social networking sites.

The goals for future work are first to make a more systematic comparison of public usage to the scientific corpus, and then second to devise an algorithm to search the dataset, find diffusion peaks, find the best fit of a Bass process to each, and return a 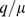 ratio. We would need to construct a critical test for a leveling-off that indicates a word has ceased to be trendy and enters the language functionally (such as *DNA* or *radio*). This would require an automated process examining large datasets, which might be an algorithm that defines the “birth” of a new word in one of two ways, either (a) the time at which the logged frequency of the word grows in ten consecutive time periods or (b) by an order of magnitude in a shorter time period (this simple pair of rules is consistent with the visual start date to within several years in almost all cases).

## Conclusions

Our goal has been to demonstrate the potential of a simple model for characterizing word-usage trends, which then can be used to inform efforts at better communication. Recognizing which words spread by diffusion, along with the ideas or metaphors they represent, can justify an information campaign shifting its focus toward social learning rather than expecting an audience to adopt a message simply because its content is objectively sound.

When one asks, “How can scientists respond?” when the public is ambivalent about climate change [9], it is tempting simply to shrug and lament that media and the public are prone to fashions, even as scientists gravitate toward consensus [8]. As Orwell [12] reminded us long ago, however, the trends of English usage might be the key to improving the politics that surround science. In a recent book [35], we discuss the example of the small Danish island of Samsø, whose inhabitants succeeded in shifting the island's energy supply from oil entirely over to renewable wind turbines, even though those cost about $1 million apiece [36]. Several key elements appear to have been pivotal in this remarkable, inspiring transformation, but for this expensive new behavior to spread, social learning was key. In small and socially cohesive Samsø communities, the project leader promoted the idea at every opportunity, from local town meetings to everyday conversations, which later became an organic component of daily conversation, as newly erected wind turbines became a highly visible part of the constructed environment [35], [36].

As we believe to be the case for words of a language, the parameters of the model can be argued to represent social versus individual decision making. As we discussed above, however, the same sorts of adoption curves can be achieved through some distribution of purely independent response times [29]. It remains for future research to attack this “identification problem” of separating actual social forces from independent forces in the observed dynamics of word usage. Of course, one means to address this is not to rely on curve fitting but to use it merely as a quantitative population-scale tool to complement qualitative local-scale investigation such as ethnography, interviews, or discourse analysis [37], [38]. Hence, the curve fitting becomes a means of presenting hypotheses for qualitative, detailed investigation, including interesting exceptions that depart from the Bass model. An example would be the “presidential” boost in Google searches for “bird flu” in November 2005 exhibited after President Bush announced a $7 billion “Bird Flu Strategy” [18], or the boost in the names associated with U.S. presidents and their family members in the year following their election [39]. Alternatively, other words have declined so sharply in time as to signify forms of censorship or sudden social inappropriateness, such as the word *slavery* after 1865 [15]. In a less dramatic sense, the residuals from our models suggest some bias against *adaptation* and 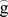
lobal in the last years of the dataset (to 2008). Though time will tell how this plays out, it demonstrates the utility of this simple model as a tool for identifying subtler trends.

## Methods

### The model

In the Bass [30] formulation of equation (1), 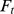 is the cumulative distribution function and 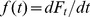 is what Bass described as the density function. The ratio 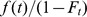, representing adoption rate as a fraction of potential adopters remaining, is known as the Bass “hazard function.” We assume the total population size is fixed at one, so that 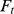 is the fraction of eventual uses of the word by time 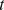, and 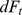 is the number of new users during 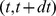. In order to predict the date of peak adoption rate, we differentiate equation (1) and obtain.
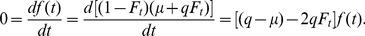
(10)


This maximum occurs at a date 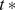 when the density 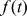 takes a maximum. At this maximum, the cumulative-adoption fraction, 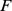, is
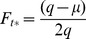
(11)


Bass [30] solved (4) and (5) and found that 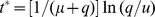.

#### Non-social version

In comparing the Bass diffusion model to the word data, we acknowledge that the parameter 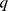 is merely reflecting frequency-dependent growth, which does not necessarily have to be “social,” as S-curves of adoption can be generated through individual learning in successive stages [29]. The full literature on discrete-choice models is beyond the scope of the current study, but to take an example, let the net cumulated utility to the usage of word 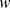 by date 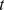 be denoted by
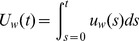
(12)


We can then apply a discrete-choice model [25], whereby the choice between using word 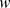 and some other word is given by
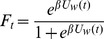
(13)


Differentiating both sides of equation (13), we obtain
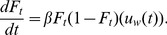
(14)


Assuming 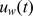 is positive and constant through time, then 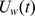 increases steadily through time and we replicate Bass diffusion, with the “individualistic” term 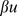 acting like the “social” 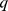 parameter in equation (1). Effectively, we have re-labeled the parameter that governs frequency-dependent growth of the word usage from “social” to “accumulated utility.” As described above, however, we feel comfortable in the specific case of this study of language use, which is inherently social, to refer to the parameter 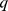 as the social parameter.

Regarding equation (2) above, in which 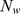 grows with time, we can follow Brock and Durlauf [26], who specify a hazard function of this sort and (dropping the covariates) arrive at the same two-parameter Bass hazard function as in equation (1) above, where 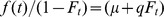. In order to be thorough with our approach of inserting equation (6) into (2) using the empirical counts of the word *the*, which dropped in relative frequency from about 6% to about 5% over three centuries, we would need to add to the RHS of equation (2) a discrete time analog of the term
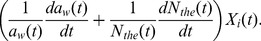
(15)


However, we can afford to neglect this entire term because (a) under the maintained hypothesis that 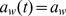 is constant for all dates 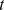, 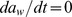, and (b) 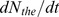 is also small, as it took centuries for *the* to decrease from 6% to 5%.

#### Noisy version

In order to introduce “noise” in both 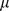 and 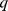 during these modeled dynamics, we introduce the noise term, 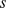, the amplitude of which is governed by 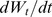, where 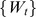 is a standardized Wiener process. We may then write
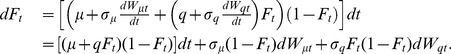
(16)


Dividing both sides of equation (16) by 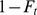, the remaining potential adoptions, we have the following for 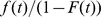, which is also known as the Bass hazard function:
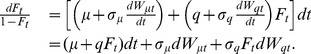
(17)


Note that if 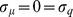, we recover the deterministic case where 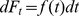 is the absolute word-adoption rate during 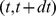 and 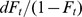 is again the Bass adoption rate per potential adoption yet to be made.

To focus first on noise in the parameter 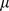, we eliminate the noise in 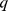 by setting 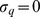. Because 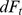 is Bass adoptions during 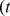, 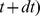, we have
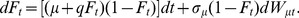
(18)


We may compute the variance of usage (ignoring the truncation issue in that 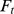 must always be positive, meaning that we must use a “truncated” normal when 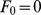 and 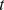 is near zero),
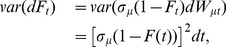
(19)where we used the basic property of standardized Wiener processes, 
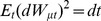
. Hence, noise in 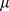 implies the variance of adoption rate, 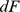, during 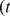, 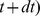 will decline as future potential adoptions, 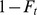, also decline. Next, we add noise in 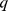, such that 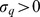 and



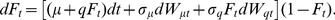
(20)Hence, 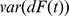 is given by
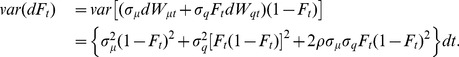
(21)


Here, 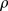 is the correlation between the noises shocking the inventors (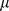 in equation (1)) and the noises shocking the imitators (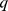 in equation (1)). The correlation between the noises and the relative sizes of the noises should differ across contexts. For parsimony, however, we set 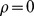. This secondary variable could be investigated in the future.

### Data

For each word in our case study, we obtained the time series of word frequencies via Google's Ngram tool from the 10 CSV data files (approximately 1 GB each) provided for 1-grams among the datasets combining both British and American English. Google distributes the 1-grams data into nine comma-separated values files, which we imported into a MySQL database. A substantial fraction of these 1-grams are not words, and we therefore removed all 1-grams consisting of commonly used symbols or numbers, as well as any 1-gram that contained the same consonant three or more times consecutively. A MySQL table was then created that contained the 1-grams that passed through the filters.

For each word we examined, one of these 10 files provides the integer number of appearances, per calendar year, in 4% of all English-language books (the data also include the number of published pages the 1-gram appeared on and the number of different books it appeared in; we do not use these measures). The 1-grams are case-sensitive, and we used the lowercase version of all words. The word counts run from about the mid-17th century to 2008. This remarkable dataset has a minor constraint in that it includes only Ngrams that appear over 40 times in the whole corpus (ngrams.googlelabs.com/datasets); this bounds the observable Zipf's Law at extremely low frequencies of occurrence, which has no effect on our observances of the top 1000 most-common words through time.

We used Java code to analyze the data in these MySQL tables of filtered and raw data. To produce the distributions of 1-gram frequencies, we first queried the raw data to produce a list of Ngrams and their frequencies for a year of interest. We then cross-referenced this with the table of filtered Ngrams to remove nonwords.

### Fitting

To test whether these words can be fitted with the simple Bass diffusion model, we estimated 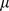, 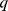, plus either 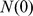 for the exponential version of equation (5) for Model 1, or 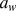 in the best fit of equation (7) for Model 2. We estimated the three parameters by applying a nonlinear fitting algorithm (“nlinfit” in MATLAB) to the word frequencies. Based on minimizing the least-squares regression between the nonlinear function and the data [14], this algorithm searches the space of parameters by iteratively refitting a weighted nonlinear regression. It bases the weight at each iteration on the residual from the previous iteration [40], which de-emphasizes the influence of outliers on the fit, and the iterations are continued until the weights converge [41].
